# Adipocyte-Specific Inhibition of *Mir221/222* Ameliorates Diet-Induced Obesity Through Targeting *Ddit4*

**DOI:** 10.3389/fendo.2021.750261

**Published:** 2022-01-03

**Authors:** Satoshi Yamaguchi, Dongxiao Zhang, Akihiro Katayama, Naoko Kurooka, Ryosuke Sugawara, Haya Hamed Hassan Albuayjan, Atsuko Nakatsuka, Jun Eguchi, Jun Wada

**Affiliations:** Department of Nephrology, Rheumatology, Endocrinology and Metabolism, Okayama University Graduate School of Medicine, Dentistry and Pharmaceutical Sciences, Okayama, Japan

**Keywords:** non-coding RNAs, microRNA, adipose tissues, Adipogenesis, mTORC1

## Abstract

MicroRNAs expressed in adipocytes are involved in transcriptional regulation of target mRNAs in obesity, but miRNAs critically involved in this process is not well characterized. Here, we identified upregulation of miR-221-3p and miR-222-3p in the white adipose tissues in C57BL/6 mice fed with high fat-high sucrose (HFHS) chow by RNA sequencing. *Mir221* and *Mir222* are paralogous genes and share the common seed sequence and *Mir221/222AdipoKO* mice fed with HFHS chow demonstrated resistance to the development of obesity compared with *Mir221/222^flox/y^*. *Ddit4* is a direct target of *Mir221* and *Mir222*, and the upregulation of *Ddit4* in *Mir221/222AdipoKO* was associated with the suppression of TSC2 (tuberous sclerosis complex 2)/mammalian target of rapamycin complex 1 (mTORC1)/S6K (ribosomal protein S6 kinase) pathway. The overexpression of miR-222-3p linked to enhanced adipogenesis, and it may be a potential candidate for miRNA-based therapy.

## Introduction

In obesity, the excess and ectopic accumulation of adipose tissue leads to the clusters of metabolic disorders such as low-grade inflammation, type 2 diabetes (T2D), dyslipidemia, hypertension, nonalcoholic fatty liver disorder (NAFLD), cardiovascular diseases (CVDs), chronic kidney disease (CKD) and cancer. As an endocrine organ, the adipose tissue communicates with other tissues by secreting peptide hormones, inflammatory cytokines, signaling lipids and miRNAs packed in exosomes (1). The alteration of expression profile of miRNA both in cytoplasm and extracellular vesicles leads to changes in the transcriptional and translational activities of the genes, which control inflammation, whole body insulin sensitivity, lipid metabolism, adipogenesis of white, beige, and brown adipose tissues (2). For instance, miR-34a secreted by mature adipocytes in exosomes were transported into macrophages, suppresses M2 polarization, and stimulates inflammatory responses by repressing Krüppel-like factor 4 (3). Lentivirus-mediated suppression of miR-34a increased the beige and brown fat formation in diet-induced obese mice by increasing fibroblast growth factor 21 and sirtuin 1 (4). The genetic ablation of miR-128-1 in mouse metabolic disease models resulted in increased energy expenditure, amelioration of diet-induced obesity and enhanced insulin sensitivity (5). In lipid metabolism, miR-425 overexpression in mice resulted in the promotion of diet-induced obesity and overexpression of miR-425 in 3T3-L1 cells accelerated adipogenesis and lipogenesis, while knock down of miR-425 remarkably enhanced lipolysis and lipid oxidation (6).

We also attempted to identify specific miRNAs critically involved in the process of obesity, we surveyed expression profile of miRNAs in liver, muscle, white adipose tissues (WATs), and sera of C57BL/6JJcl mice fed with standard (STD) and high fat-high sucrose (HFHS) chow by RNA sequencing (GSE61959) (7). miR-221-3p and miR-222-3p are highly up-regulated in epididymal adipose tissues in mice fed with high fat high sucrose (HFHS). miR-221 and miR-222 both locate in the close proximity on the X chromosome and share the identical seed sequence (8). In meta-analysis, circulating miR-221-3p was reduced in T2D, while miR-222-3p was elevated in obesity and T2D (9). Although miR-221-3p and miR-222-3p are differentially expressed in sera and adipose tissues, the functional *in vivo* experiments to elucidate the roles of *Mir221* and *Mir222* in obesity has not been reported. Here, we investigated the adipocyte specific *Mir221* and *Mir222* knockout (*Mir221/222AdipoKO*) mice fed with HFHS chow, and they were protected from the development of obesity by targeting DDIT4 (DNA damage inducible transcript 4)/TSC2 (tuberous sclerosis complex 2)/S6K (ribosomal protein S6 kinase) pathway.

## Materials And Methods

### Animal Models

We obtained Mir 221 KO cond (*Mir221* ^tm2^; EMMA ID, EM:05507) from EMMA (the European Mouse Mutant Archive) and Helmholz Zentrum München (Neuherberg, Germany). The targeting vector cassette composed of *Neo* flanked by FRT and distal loxP sites was inserted outside the genomic region encoding *Mir221* and *Mir222* precursors. *Neo* was removed by Flp in the conditional KO allele of Mir 221 KO cond. The conditional KO allele was confirmed by primer pair; 1544_29 (5’- GCT CTG TTT TCC TAA GTG ATG G-3’) and 1544_30 (5’-CTG ACA GGA AGT AAA TCA TCT TAG C-3’). The expected fragments were 265 bp in wild and 384 bp in conditional KO allele. We also used B6.FVB-Tg (Adipoq-cre)1Evdr/J to produce *Mir221/222AdipoKO* mice by crossing to Mir 221 KO cond. Adipoq-cre transgene was screened by primers, Forward (5'-AGC GAT GGA TTT CCG TCT CT-3') and Reverse (5'-CAC CAG CTT GCA TGA TCT CC-3'). The primers, oIMR7338 (5’-CTA GGC CAC AGA ATT GAA AGA TCT-3’) and oIMR7339 (5’-GTA GGT GGA AAT TCT AGC ATC ATC C-3’), were used for internal positive control. The expected sizes for transgene and internal positive control were 200 bp and 324 bp, respectively. By crossing male Tg (Adipoq-cre) and female *Mir221* ^tm2^ */y* C57BL/6JJcl mice, we generated male *Mir221/222* ^flox/y^ and male *Mir221/222AdipoKO* littermates.

Five-week-old mice were randomly assigned to standard diet (STD) group (MF, Oriental Yeast, Japan) or high fat high sucrose diet (HFHS) group (D12331, Research Diets, New Brunswick, NJ). The detailed formulation of STD (10) (https://www.oyc.co.jp/bio/LAD-equipment/LAD/ingredient.html and https://www.oyc.co.jp/bio/LAD-equipment/LAD/rodents.html) and HFHS (https://www.eptrading.co.jp/service/researchdiets/pdf/D12328-D12331.pdf) diets are shown in **Supplementary Table 1**. At 22 weeks of age, we obtained various organs and they were subjected to following experiments.

### 3T3-L1 Cell Cultures

3T3-L1 pre-adipocytes were cultured in Dulbecco’s modified eagle’s medium (DMEM, 2124951, Gibco). On day 0, the cells were treated with the differentiation media; DMEM supplemented with 10% FBS, 10 µg/ml insulin (I1882, Sigma), 1 µM DEX (D2915, Sigma) and 0.5 mM IBMX (I5879, Sigma). Then, the media were changed to DMEM supplemented with 10 µg/ml insulin and 10% FBS on day 2 and cultured for 10 or 20 days. Undifferentiated 3T3-L1 cells were subjected to Lentiviral miRNA expression, Lentiviral miRNA inhibition studies, and luciferase reporter assays.

### Human Serum Samples

Human serum samples were collected from 69 patients with type 2 diabetes in Okayama University Hospital and 45 subjects with normal fasting glucose (NGT).

### Insulin Tolerance Test and Glucose Tolerance Test (ITT and GTT)

The 13-week-old mice were fasted for 16 hours in GTT and for 3 hours in ITT. They were then intraperitoneally injected with glucose solution (1 mg/g body weight) and human insulin (1 unit/kg in HFHS groups and 0.75 unit/kg in STD groups) for GTT and ITT, respectively.

### Basal Metabolic Rate, Locomotor Activity, and Food Intake

At 18 weeks of age, O_2_/CO_2_ metabolism measuring system (MK-5000, Muromachi Kikai, Tokyo, Japan) were used to quantify oxygen consumption rate and carbon dioxide production for the estimation of V̇O2 and respiratory quotient (RQ). The locomotor activity was recorded for 24 hours by the frequency of interrupting an infrared sensor (ACTIMO-100, Shinfactory, Fukuoka, Japan). Daily food intake was measured and calculated; daily food intake [g/day/body weight (BW)] = [initial food weight (g) – leftover food weight (g)]/measurement period (days)/BW (g). The 6-10 mice in each experimental group were examined.

### Reverse Transcription-Quantitative Polymerase Chain Reaction (RT-qPCR)

RNAs were extracted from frozen tissues and cultured 3T3-L1 cells with RNeasy Mini kit (74106, Qiagen). The QIAamp Circulating Nucleic Acid Kit (Qiagen) were used for the isolation of total RNAs from serum. For gene expression analyses, cDNAs were prepared with High-Capacity RNA-to-cDNA Kit (Thermo Fisher Scientific). TaqMan gene expression primers, *Ddit4* (Mm00512504_g1), *Rplp0* (Mm00725448_s1), *Rn18s* (Mm03928990_g1), *Adipoq* (Mm00456425_m1), *Lep* (Mm00434759_m1), *Lpl* (Mm01345523_m1), *Srebf1* (Mm00550338_m1), *Cebpa* (Mm00514283_s1), *Fabp4* (Mm00445878_m1), *Pparg* (Mm01184322_m1), *Lipe* (Mm00495359_m1), *Pnpla2* (Mm00503040_m1), *Ucp1* (Mm01244861_m1), *Cox8b* (Mm00432648_m1), *Prdm16* (Mm00712556_m1), *Cidea* (Mm00432554_m1), *Ppargc1a* (Mm01208835_m1), *G6pc* (Mm00839363_m1), *Gck* (Mm00439129_m1), *Fasn* (Mm00662319_m1), *Ppara* (Mm00440939_m1), *Il6* (Mm00446190_m1), *Ifng* (Mm01168134_m1), *Tnf* (Mm00443258_m1), *Il1b* (Mm01336189_m1) were used (Thermo Fisher Scientific). For miRNA expression studies, cDNAs were prepared from total RNAs by TaqMan MicroRNA Reverse Transcription Kit (Life Technologies). MicroRNA primers, *mmu-miR-222-3p* (CTAAJ3), *hsa-miR-221-3p* (000524), *hsa-miR-222-3p* (002276), snoRNA202 (001232), snoRNA234 (001234), and cel-miR-39 (000200) were used (Thermo Fisher Scientific). Rplp0, Rn18s, snoRNA202, snoRNA234, and cel-miR-39 were served as the invariant controls. The RT-qPCR was performed using TaqMan Universal PCR Master mix II (no UNG) at a StepOne Plus Real-Time PCR system. The quantification was performed by the 2^−ΔΔCT^ analysis method.

### Cloning of Mir221 Host Gene (*Mir221hg*)

Long non-coding RNA, *Mir221hg*, was cloned using SMARTer RACE 5’/3’ Kit (634858, Clontech). 5’- and 3’-RACE-Ready cDNAs from epididymal adipose tissues poly A^+^ RNA were prepared by SMARTScribe Reverse Transcriptase. 5’- and 3’-RACE-Ready cDNAs were amplified by PCR using 5’ GSP (5’-GAT TAC GCC AAG CTT CCA GCA GAC AAT GTA GC TGT TGC-3’) and 3’ GSP (5’-GAT TAC GCC AAG CTT TCC AGG TCT GGG GCA TGA ACC TG-3’), respectively. Furthermore, nested PCR was performed using 5’ NGSP (5’-GAT TAC GCC AAG CTT GTA TGC CAG GTT CAT GCC CCA GAC-3’) and 3’ NGSP (5’-GAT TAC GCC AAG CTT GCA ACA GCT ACA TTG TCT GCT GG-3’). PCR products were analyzed by agarose/EtBr gel and purified by NucleoSpin Gel and PCR Clean-Up Kit. The purified RACE products were subcloned into pRACE vector by In-Fusion Cloning (Clontech). Independent 4 clones of both 5’- and 3’-RACE were sequenced.

### Western Blot Analysis

The epididymal fat tissues from 22-week-old mice and cultured 3T3-L1 cells were homogenized in RIPA lysis buffer (radioimmunoprecipitation buffer) plus protease inhibitors. The samples were boiled in SDS-PAGE loading buffer, separated on 12% Mini-PROTEAN TGX Precast Protein Gels (Bio-Rad), and transferred to a PVDF Blotting Membrane (cytiva). After blocking with 5% nonfat milk for 1 hour at room temperature (RT), the blots were incubated with REDD-1/DDIT4 Antibody, rabbit polyclonal (ab106356, RRID: AB_10864294), C/EBPα Antibody, rabbit polyclonal (2295, RRID: AB_10692506), PPARγ (D69) Antibody, rabbit polyclonal (2430, RRID: AB_823599), mTOR (7C10) Rabbit mAb (2983, RRID: AB_2105622), Phospho-mTOR (Ser2448) (D9C2) XP Rabbit mAb (5536, RRID: AB_10691552), Akt Antibody, rabbit polyclonal (9272, RRID: AB_329827), Phospho-Akt (Thr308) Antibody, rabbit polyclonal (9275, RRID: AB_329828), p70 S6 Kinase (49D7) Rabbit mAb (2708, RRID: AB_390722), Phospho-p70 S6 Kinase (Thr389) Antibody, rabbit polyclonal (9205, RRID: AB_330944), Tuberin/TSC2 Antibody, rabbit polyclonal (3612, RRID: AB_2207804), Phospho-Tuberin/TSC2 (Thr1462) Antibody, rabbit polyclonal (3611, RRID: AB_329855, Cell Signaling Technology) overnight at 4°C. GAPDH (D16H11) XP Rabbit mAb (HRP Conjugate) (8884, RRID: AB_11129865) was used as a loading control (Cell Signaling Technology). After washing three times with Tris-buffered saline (TBS), the blots were incubated with ECL Donkey Anti-Rabbit IgG, HRP-Conjugated Antibodies (NA934V, GE healthcare Life science, 1:10000) at RT for 1 hour. The blots were developed with Pierce ECL Western Blotting Substrate (TE261327, Thermo Fisher Scientific). The chemiluminescence was analyzed using ImageQuant LAS-4000 mini (FUJIFILM).

### Morphometric Analysis for Adipocyte Size

Epidydimal adipose tissues were fixed by 10% formalin, embedded with paraffin. The 5-μm paraffin sections were prepared and stained with PAS. The images were captured using an Olympus BX51 microscope. The size of the adipocytes was analyzed by Keyence Hybrid cell count software. Epidydimal adipose tissues were taken from 3-5 individual animals from each experimental group.

### Isolation of Stromal Vascular Fraction (SVF) From White Adipose Tissues

SVF was isolated from epididymal adipose tissue of 24-week-old mice. Briefly, fresh mouse epididymal fat pads were minced and digested with collagenase type 1 (CLSS1, Worthington) in HBSS containing 10% FBS for 45 minutes at 37°C. The mixture was filtered through a nylon mesh (100 μm), then centrifuged at 400 g for 1 minute. The adipocyte fraction was obtained from the supernatant, they were again centrifuged at 800 g for 10 minutes, and the SVF was obtained from the pellet.

### Identification of *Mir221/222* Target mRNAs

The mRNA microarray was performed by GeneChip Mouse Gene 2.0 array using total RNA of epidydimal fat obtained from 16-week-old mice (1 animal from each group) and analyzed by Filgen (Nagoya, Japan). The raw data are available in Gene Expression Omnibus (GEO; https://www.ncbi.nlm.nih.gov/geo/) (GSE163921). TargetScan (http://www.targetscan.org/vert_72/), miRDB (http://www.mirdb.org/), and DIANA-microT v5.0 (https://bio.tools/DIANA-microT) and were used to identify potential target genes for *Mir221/222*.

### Lentiviral miRNA Expression and Lentiviral miRNA Inhibitor

Lentiviral transduction using pLV-miRNA and pLV-miR-Locker system to 3T3-L1 cells were performed according to the manufacturer’s manual (Biosettia). After transformation to *E. coli* JM109 cells, pLV-[mmu-mir-221] plasmid (mir-p177m, Biosettia), pLV-[mmu-mir-222] plasmid (mir-p178m, Biosettia), pLV-[mmu-mir-221-3p] locker plasmid (mir-mp0337, Biosettia), pLV-[mmu-mir-222-3p] locker plasmid (mir-mp0339, Biosettia), pLV-[mir-control] plasmid (mir-p000, Biosettia), pLV-miR-locker control plasmid (mir-locker-ctrl, Biosettia), pMDLg/pRRE (12251, Addgene), pRSV/Rev (12253, Addgene), and pMD2.G (12259, Addgene) were isolated with EndoFree Plasmid Maxi Kit (12362, Qiagen). 293T cells (10 × 10^7^/5 mL) were transfected with each pLV plasmid, pMDLg/pRRE, pRSV/Rev, pMD2.G, Lipofectamine LTX, and 1.5 mL Opti-MEM. The supernatants were collected after 48 hours after transfection. 3T3-L1 cells were transduced with lentivirus stock in complete media containing 10 μg/mL polybrene for 12 hours, replaced with fresh complete medium

### Luciferase Reporter Assay

To quantitatively evaluate miRNA activity on cloned miRNA target sequence from 3’-untranslated region (3’-UTR) of *Ddit4*, pmirGLO dual luciferase miRNA Target expression vector (E1330, Promega) was used. Firstly, the pmirGLO plasmid was linearized by double digestion with *Xho*I and *Sal*I. The cDNA of *Ddit4* wild type (WT) 3′-UTR was amplified by PCR and ligated with CIP treated pmirGLO Vector. The primers are Forward-*Xho*I-3’UTR-*Ddit4*: 5’-GGG GGG CTC GAG CAG CTG CTC ATT GAA GAG TG-3′, and Reverse-*Sal*I-3’UTR-*Ddit4*: 5’-GGG GGG GTCGAC CAA ACC AAC AGA GGA GAC AG-3′. pmirGLO*-Ddit4* MT 3’-UTR was prepared by site directed mutagenesis by PCR using primers; Forward-MT-Seed-*Ddit4*: 5’-CTG GAT GTG TAT CTG CAT GTA C-3’ and Reverse-MT-Seed-*Ddit4*: 5’-GTA CAT GCA GAT ACA CAT CCA G-3’. The seed sequence “CGATGTA” was mutated to “CGTCTA”. After transformation to *E. coli* JM109 cells, pmirGLO*-Ddit4* WT 3’-UTR, pmirGLO*-Ddit4* MT 3’-UTR, and pmirGLO no-insert control plasmids were isolated with EndoFree Plasmid Maxi Kit (12362, Qiagen). 3T3-L1 cells were seeded at a density of 120,000 cells/mL, then co-transfected with Syn-mmu-miR-221-3p (MIMAT0000669, Qiagen), Syn-mmu-miR-222-3p (MIMAT0000670, Qiagen), negative control siRNA (1027280, Qiagen), inhibitor negative control (1027271, Qiagen), pmirGLO*-Ddit4* WT 3’-UTR, pmirGLO*-Ddit4* MT 3’-UTR, and pmirGLO no-insert control plasmids. Twenty-four hours after transfection, the cells were analyzed to measure luciferase activities using the Dual-Glo Luciferase Assay System and a GloMax 20/20 luminometer (Promega).

### Statistical Analysis

All values were represented as the mean ± standard deviation (SD). Statistical analyses were conducted using IBM SPSS Statistics 23 and GraphPad Prism (version 8.0). Independent *t*-test, Mann-Whitney’s U test, and one-way ANOVA with Tukey test was used to determine the differences. For correlation, non-parametric Spearman r coefficient was used. p<0.05 was considered statistically significant.

## Results

### *Mir221/222AdipoKO* Mice Are Resistant to Diet-Induced Obesity

To identify miRNAs critically involved in the disease process of obesity and type 2 diabetes (T2D), we performed miRNA profiling of serum, liver and epididymal fat tissues in C57BL/6JJcl mice fed with standard (STD) and high fat-high sucrose (HFHS) chow. The Illumina RNA sequencing data (Gene Expression Omnibus number GSE61959) demonstrated that the read numbers of *Mir221* and *Mir222* were 5.7 and 8.2-fold up-regulated in epididymal adipose tissues in HFHS group compared with STD group (**Supplementary Table 2**). *Mir221* and *Mir222* are paralog genes located in proximity on X chromosome and they share identical seed sequence. To further investigate the role of *Mir221/222* in obesity and diabetes, we crossed male Tg (Adipoq-cre) mice and female *Mir221^tm2/y^* C57BL/6JJcl mice and generated male *Mir221/222^flox/y^* and male *Mir221/222AdipoKO* littermates.

**Figure 1:**
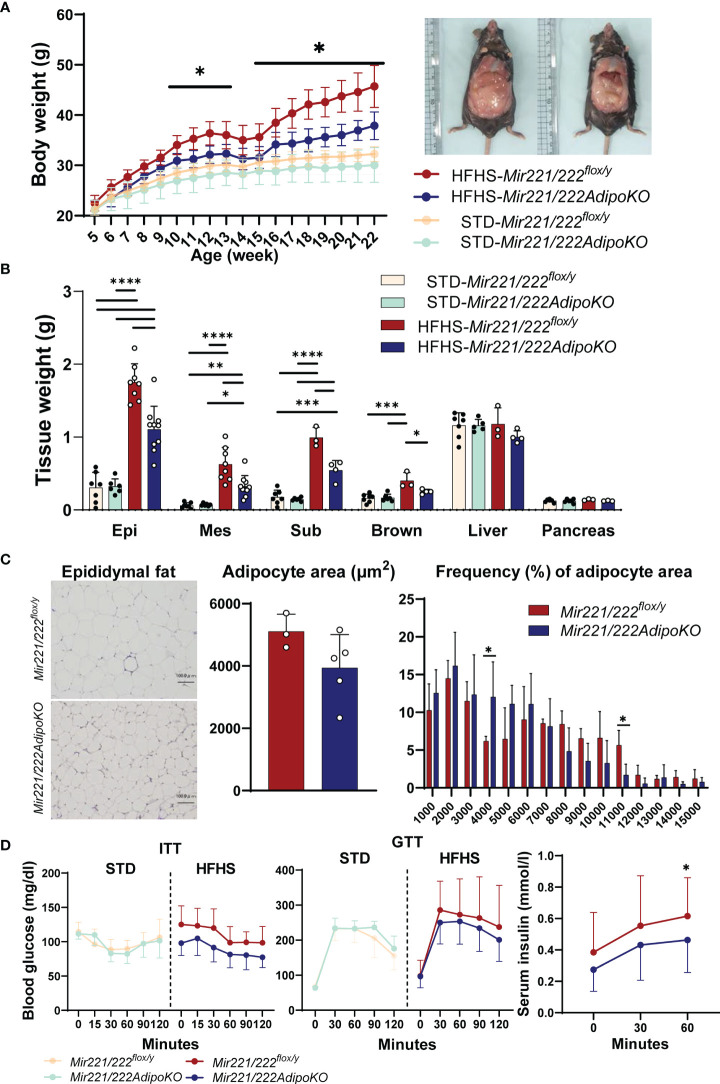
The metabolic phenotypes of *Mir221/222^flox/y^* and *Mir221/222AdipoKO* male mice fed with high fat high sucrose (HFHS) or standard (STD) chow. **(A)** Body weight of *Mir221/222^flox/y^* and *Mir221/222AdipoKO* mice fed with HFHS (n=7) or STD chow (n=6), respectively. **(B)** Tissue weight of *Mir221/222^flox/y^* and *Mir221/222AdipoKO* mice fed with HFHS or STD chow at 24 weeks of age. (Epi, epididymal; Mes, mesenteric; Sub, inguinal; Brown, Brown adipose tissues) **(C)** Adipocyte area in epididymal adipose tissues of *Mir221/222^flox/y^* (n=3) and *Mir221/222AdipoKO* (n=5) mice fed with HFHS or STD. Quantitative analyses were carried out on PAS-stained paraffin sections. **(D)** Insulin tolerance test (ITT) in Mir221/222flox/y (n=7) and Mir221/222AdipoKO (n=6). Glucose tolerance test (GTT) in Mir221/222flox/y fed with HFHS (n=13) or STD chow (n=7) and Mir221/222AdipoKO fed with HFHS (n=16) or STD chow (n=6). Data shown as mean ± SD and analyzed by one-way ANOVA with Tukey test in **(A, B)**, and Mann-Whitney’s U test in **(C, D)** (*p<0.05; **p<0.01; ***p<0.001; ****p<0.0001).

Body weight of *Mir221/222AdipoKO* mice fed with HFHS chow was significantly reduced compared with *Mir221/222^flox/y^* mice (**Figure 1A**). The weight of epididymal, mesenteric, subdermal, and brown fat was also reduced in *Mir221/222AdipoKO* mice (**Figure 1B**). The *Mir221/222^flox/y^* and *Mir221/222AdipoKO* mice fed with STD chow demonstrated no significant differences in their body and tissue weight (**Figures 1A** **, B**). The average size of adipocytes in epididymal adipose tissues derived from *Mir221/222AdipoKO* mice fed with HFHS chow was smaller compared with *Mir221/222^flox/y^* mice (**Figure 1C**). To investigate glucose homeostasis, we performed insulin tolerance test (ITT) and glucose tolerance test (GTT). The blood glucose levels of *Mir221/222AdipoKO* mice fed with HFHS chow were reduced in GTT and ITT compared with *Mir221/222^flox/y^*, but they did not reach significant differences (**Figure 1D**). The serum insulin concentrations of *Mir221/222AdipoKO* mice were significantly lower than *Mir221/222^flox/y^*, suggesting the improvement of insulin sensitivity in *Mir221/222AdipoKO* mice (**Figure 1D**). To investigate whether reduced adiposity in *Mir221/222AdipoKO* mice was due to changes in energy expenditure or energy intake, we measured basal metabolic rates, locomotor activity and food intake. Basal metabolic rate such as respiratory quotient and oxygen consumption were not altered between *Mir221/222^flox/y^* and *Mir221/222AdipoKO* mice fed with HFHS chow (**Supplementary Figures 1A, B**). The locomotor activity was recorded for 24 hours, most of the activities were observed during the dark phase. Increased activity was observed in *Mir221/222AdipoKO* mice under HFHS chow compared with *Mir221/222^flox/y^* during light (0.944 ± 0.309 *vs* 1.60 ± 0.764 counts/min, p=0.036) and dark (6.67 ± 4.38 *vs* 7.94 ± 4.38 counts/min, p=0.497) periods (**Supplementary Figure 1C**). Food consumptions were not altered in *Mir221/222AdipoKO* and *Mir221/222^flox/y^* fed with HFHS (**Supplementary Figure 1D**). Under HFHS chow, serum leptin concentrations were significantly reduced in *Mir221/222AdipoKO* compared with *Mir221/222^flox/y^* (24.5 ± 2.35 *vs* 21.5 ± 1.85 ng/ml, p=0.043), while serum adiponectin concentrations were not altered (**Supplementary Figures 1E, F**). In quantitative RT-PCR in epididymal adipose tissues, mRNA expression of *Lep* was significantly reduced in *Mir221/222AdipoKO*. The genes related to adipogenesis, such as *Pparg*, *Cebpa*, *Ppargc1* and *Prdm16*, were not altered in *Mir221/222AdipoKO* (**Supplementary Figure 2**).

### Expression *Mir221* and *Mir222* Are Highly Induced by HFHS Chow Feeding in Mature Adipocytes From Epidydimal Adipose Tissue

**Figure 2:**
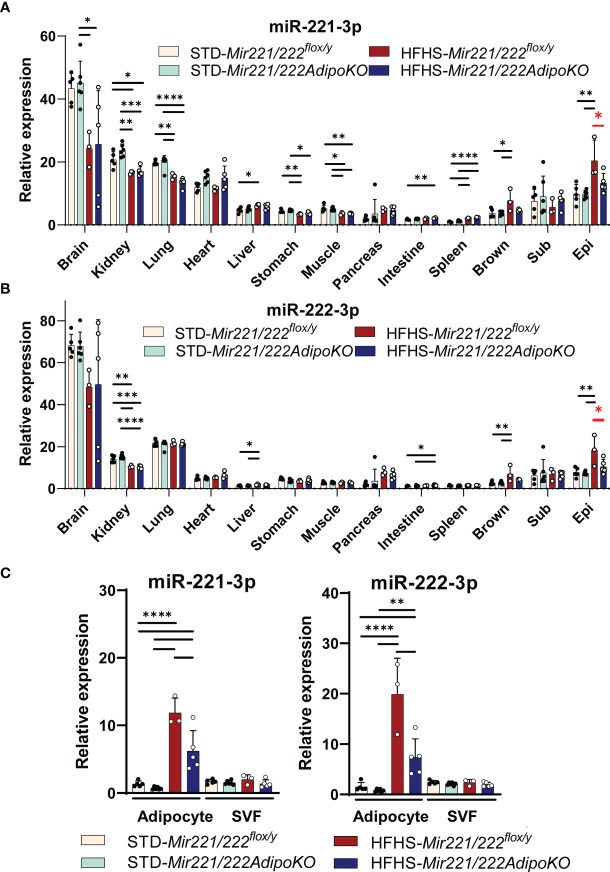
Expression of Mir221 and Mir222 in *Mir221/222^flox/y^* and *Mir221/222AdipoKO* male mice fed with high fat high sucrose (HFHS) or standard (STD) chow. **(A, B)** In various tissues, the expression of miR-221-3p and miR-222-3p is normalized by snoRNA202 and snoRNA234. The HFHS-induced up-regulation of miR-221-3p and miR-222-3p in epididymal adipose tissues was reversed in *Mir221/222AdipoKO* (red asterisks). (Epi, epididymal; Mes, mesenteric; Sub, inguinal; Brown, Brown adipose tissues) **(C)** Serum concentration of miR-221-3p and miR-222-3p. HFHS *Mir221/222^flox/y^* (n=3), HFHS *Mir221/222AdipoKO* (n=5), STD *Mir221/222^flox/y^* (n=5) and STD *Mir221/222AdipoKO* mice (n=6). Data shown as mean ± SD and analyzed by one-way ANOVA with Tukey test (*p<0.05; **p<0.01; ***p<0.001; ****p<0.0001).

We investigated the expression of *Mir221* and *Mir222* in various organs. Both miR-221-3p and miR-222-3p were abundantly expressed in brain, kidney, and lung in *Mir221/222^flox/y^* and they were down-regulated or not altered by the HFHS feeding compared with STD chow. In contrast, both miR-221-3p and miR-222-3p were significantly upregulated in the epidydimal fat tissues, and such upregulation was canceled in the epidydimal fat tissues of *Mir221/222AdipoKO* fed with HFHS chow (**Figures 2A** **, B**). Next, we investigated the localization of *Mir221* and *Mir222* in the cell fractions of epididymal adipose tissues. Both miR-221-3p and miR-222-3p were significantly induced by HFHS chow in mature adipocytes of *Mir221/222^flox/y^*, while the upregulation was significantly reversed in the mature adipocyte of *Mir221/222AdipoKO* fed with HFHS chow. In contrast, miR-221-3p and miR-222-3p were not significantly upregulated in the stromal vascular fraction (SVF) from *Mir221/222^flox/y^* fed with HFHS chow (**Figure 2C**). We also assessed the expression of miR-221-3p and miR-222-3p during 3T3-L1 adipocyte differentiation. Both miR-221-3p and miR-222-3p continuously down-regulated after the induction of adipocyte differentiation (**Supplementary Figure 3A**). Finally, we assessed the serum concentrations of mmu-miR-221-3p and mmu-miR-222-3p by quantitative PCR and they were not altered in *Mir221/222^flox/y^* and *Mir221/222AdipoKO* fed with STD and HFHS chow, suggesting serum mature forms of *Mir221* and *Mir222* were derived from various organs but not exclusively from adipose tissues (**Supplementary Figure 4A**). In human samples from the subjects with normal glucose tolerance (NGT, n=45) and impaired glucose tolerance (IGT, n=69), hsa-miR-221-3p and hsa-miR-222-3p demonstrated negative (R^2^ = 0.1311, p<0.0001) and positive correlations (R2 = 0.03841, p=0.0441) with HbA1c levels, respectively (**Supplementary Figures 4B, C**).

### Identification of *Mir221hg* and Its Expression

The miRNA genes are classified as intergenic and intragenic miRNAs, and the intragenic miRNAs and related host genes share the common transcriptional regulation. The long non-coding RNA (lncRNA), namely mir-221 host gene (MIR221HG), has been reported in bovine, which inhibits the adipocyte differentiation in cultured cells (11). Thus, we performed 5’ and 3’ rapid amplification of cDNA ends (5’/3’RACE) using poly A^+^ RNA purified from epidydimal fat tissues of C57BL/6JJcl mice and cloned 1537 bp single exon lncRNA (*Mir221hg*, MW581002) which overlap *Mir221* (**Supplementary Figure 5A**). Next, we designed primers for quantitative PCR of *Mir221hg* and evaluated gene expression in various tissue. *Mir221hg* was abundantly expressed in brain in *Mir221/222^flox/y^* and they were upregulated in brain, heart, liver, intestine, spleen by the HFHS feeding compared with STD chow. The expression of *Mir221hg* was rather low in various adipose tissues and its lncRNA expression was not changed in *Mir221/222AdipoKO* fed with STD and HFHS chow (**Supplementary Figures 5B, C**). Although *Mir221hg* lncRNA may be functional as a reservoir for miR-221-3p, the role of *Mir221hg* lncRNA in adipose tissues was limited in our experiments.

### miR-221-3p and miR-222-3p Target *Ddit4*

**Figure 3:**
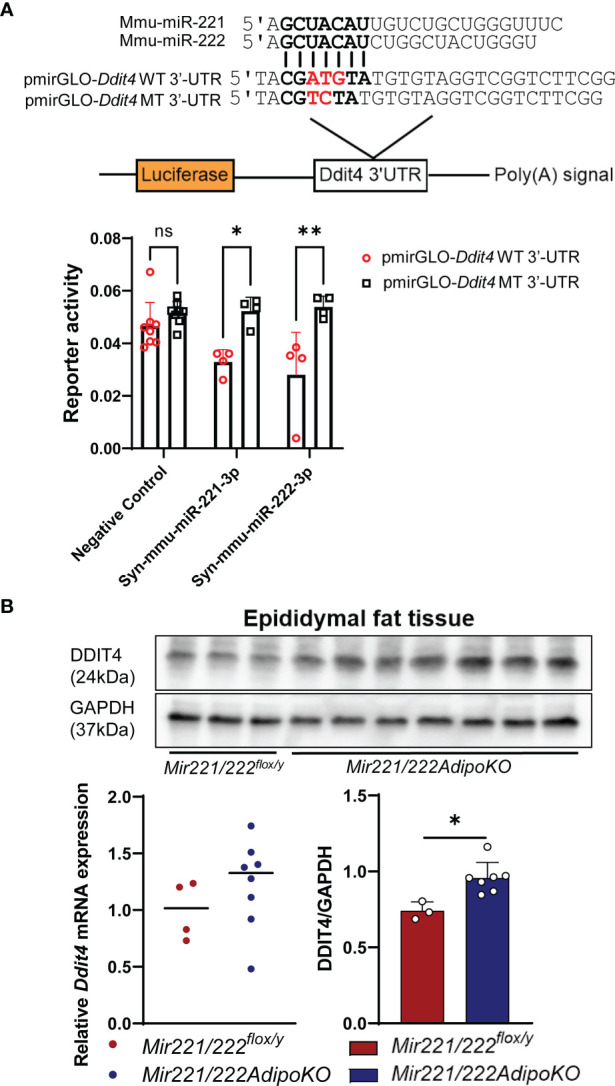
Dual-luciferase reporter assay for *Ddit4* 3’UTR and expression of *Ddit4* in mice. **(A)** Dual-luciferase reporter assay using pmirGLO*-Ddit4* WT 3’-UTR, pmirGLO*-Ddit4* MT 3’-UTR, and pmirGLO no-insert control plasmids. 3T3-L1 cells were co-transfected with Syn-mmu-miR-221-3p (n=4), Syn-mmu-miR-222-3p (n=4), negative control siRNA (n=8). (ns, not significant). **(B)** Quantitative RT-PCR for *Ddit4* in epididymal adipose tissues of *Mir221/222^flox/y^* (n=4) and *Mir221/222AdipoKO* (n=8) mice fed with high fat high sucrose (HFHS). Western blot analyses for DDIT4 and GAPDH (glyceraldehyde-3-phosphate dehydrogenase) in epididymal adipose tissues of *Mir221/222^flox/y^* (n=3) and *Mir221/222AdipoKO* (n=7) mice fed with high fat high sucrose (HFHS). Data shown as mean ± SD and analyzed by Mann-Whitney’s U test (*p<0.05; **p<0.01).

To investigate the mechanism for the resistance to diet-induced obesity in *Mir221/222AdipoKO*, we attempted to identify the potential target genes for miR-221-3p and miR-222-3p. We performed mRNA profiling by GeneChip Mouse Gene 2.0 array using total RNA purified from epidydimal fat tissues of *Mir221/222^flox/y^* fed with STD or HFHS, *Mir221/222AdipoKO* fed with STD or HFHS chow (**Supplementary Tables 3**–**5**). Then, we made the list of 355 target genes commonly predicted by TargetScan, miRDB, and DIANA-microT, and their seed sequences were conserved in both human and mouse genome (**Supplementary Table 6**). By searching upregulated genes in *Mir221/222AdipoKO* compared with *Mir221/222^flox/y^* in both STD and HFHS fed groups, we identified that DNA-damage-inducible transcript 4 (*Ddit4*) demonstrated 1.9-fold and 2.58-fold upregulation in *Mir221/222AdipoKO* fed with HFHS and STD chow, respectively (**Supplementary Table 7**). To evaluate miRNA activity of miR-221-3p and miR-222-3p by the binding to 3’-untranslated region (3’-UTR) of *Ddit4*, we performed luciferase reporter assay in 3T3-L1 cells. We cloned 3’-UTR regions of *Ddit4* gene and prepared the wild-type reporter vector, pmirGLO-*Ddit4* WT 3’-UTR, and the mutant vector, pmirGLO-*Ddit4* MT 3’-UTR, in which mutagenesis was induced on the seed sequence binding site (**Figure 3A**). The luciferase reporter vectors were co-transfected with Syn-mmu-miR-221-3p or Syn-mmu-miR-222-3p or negative control siRNA in 3T3-L1 cells. As a result, reporter activity of pmirGLO-*Ddit4* WT 3’-UTR were significantly inhibited in the presence of both Syn-mmu-miR-221-3p and Syn-mmu-miR-222-3p. In contrast, Syn-mmu-miR-221-3p or Syn-mmu-miR-222-3p had almost no effect on the reporter activity of pmirGLO-*Ddit4* MT 3’-UTR (**Figure 3A**). Next, we checked the expression of *Ddit4* in the epidydimal adipose tissues of *Mir221/222^flox/y^* and *Mir221/222AdipoKO* fed with HFHS by quantitative PCR and Western blot. Although the mRNA expression of *Ddit4* was increased in *Mir221/222AdipoKO* without statistical significance, DDIT4 protein expression was significantly upregulated in the *Mir221/222AdipoKO* compared with *Mir221/222^flox/y^* (**Figure 3B**), suggesting the possible involvement of translational repression by miR-221-3p and miR-222-3p.

### Mir221/Mir222 Promotes Adipogenesis by *DDIT4*-Mediated Inhibition of mTORC1

**Figure 4:**
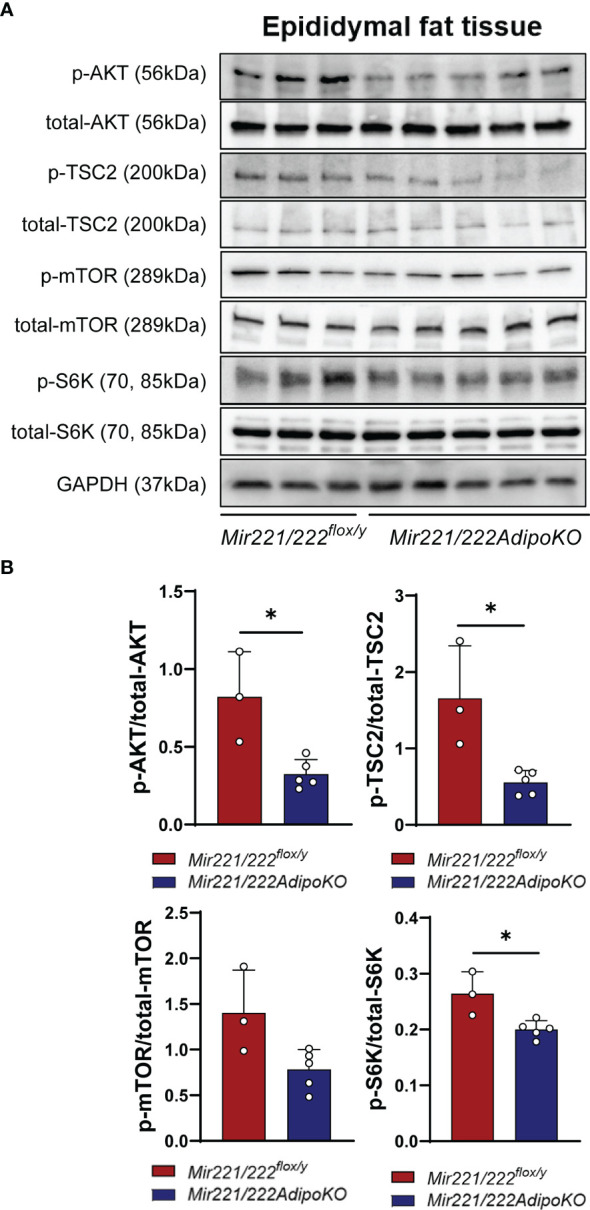
Western blot analyses. **(A)** Western blot analyses for AKT (protein kinase B), TSC2 (tuberous sclerosis complex 2), mTOR (mammalian target of rapamycin) and S6K (ribosomal protein S6 kinase) in *Mir221/222^flox/y^* (n=3) and in epididymal adipose tissues of *Mir221/222AdipoKO* (n=5) male mice fed with high fat high sucrose (HFHS). Activated form of p-AKT and Inactivated form of p-TSC2 (Thr-1462) and activated form of p-S6K are shown. **(B)** Densitometric analyses of Western blots. Data shown as mean ± SD and analyzed by Mann-Whitney’s U test (*p<0.05).

DDIT4 is known to activate tuberous sclerosis complex 1 (TSC1)/TSC2 complex by the inhibition of AKT (protein kinase B) and the release of 14-3-3 from TSC2 (12). It further negatively regulates mammalian target of rapamycin complex 1 (mTORC1) (13). Increased activity of the mTORC1 signaling has been associated with obesity (14), and the knockout mice for the mTORC1 downstream ribosomal protein S6 kinase (S6K) are protected against diet-induced-obesity (15). Thus, we further checked the inactivated form of p-TSC2 (Thr-1462) and activated form of p-S6K (16) in the epidydimal adipose tissues of *Mir221/222^flox/y^* and *Mir221/222AdipoKO*. After overnight fasting, the mice were injected intraperitoneally with insulin and the epidydimal fat tissues were harvested at 7 min after insulin injection. In *Mir221/222AdipoKO* mice, both p-AKT and p-TSC2 (Thr-1462) were significantly reduced, and accordingly p-S6K was also significantly reduced (**Figures 4A, B**). p-mTOR was reduced in *Mir221/222AdipoKO* mice, however, it did not reach the statistically significant differences (**Figures 4A, B**).

**Figure 5:**
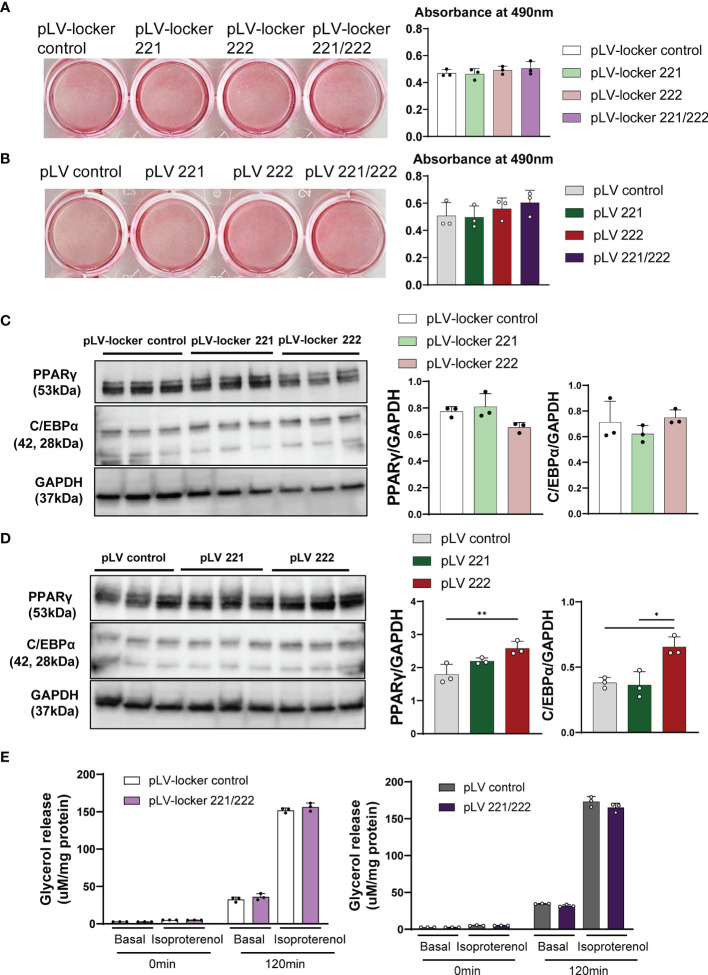
Adipogenesis, lipogenesis and lipolysis in 3T3-L1 cells. Lentiviral vectors were transduced to 3T3-L1 cells at 2 days before the induction of differentiation and cultured for 7 days. **(A)** Oil-red O staining of differentiated 3T3-L1 cells treated with pLV-locker control, pLV-locker 221, pLV-locker 222, and pLV-locker 221/222. **(B)** Oil-red O staining of differentiated 3T3-L1 cells treated with pVL control, pLV 221, pLV 222, and pLV221/222. **(C)** Western blot analysis of PPARγ in differentiated 3T3-L1 cells treated with pLV-locker control, pLV-locker 221, pLV-locker 222, and pLV-locker 221/222. **(D)** Western blot analysis of PPARγ in differentiated 3T3-L1 cells treated with pVL control, pLV 221, pLV 222, and pLV221/222. **(E)** Lipolysis assay in 3T3-L1 cells stimulated with isoproterenol. Differentiated 3T3-L1 cells were treated with pLV-locker 221/222 and pLV 221/222. Data shown as mean ± SD and analyzed by one-way ANOVA with Tukey test (*p<0.05; **p<0.01).

Since mTOR has been shown to positively regulate adipogenesis and lipogenesis while inhibiting lipolysis, fatty acid oxidation and ketogenesis (17), we performed adipogenesis and glycerol assays in 3T3-L1 adipocytes. We constructed lentiviral vector expressing miRNA inhibitors (pLV-locker 221, pLV-locker 222, pLV-locker control) and miRNA mimics (pLV 221, pLV 222, pLV control). The efficiency for inhibitors and mimics was confirmed in 3T3-L1 cells at 3-7 days after the transduction (**Supplementary Figures 3B, C**). Lentiviral vectors were transduced to 3T3-L1 cells at 2 days before the induction of differentiation and cultured for 7 days. The pLV-locker 221, pLV-locker 222, and pLV-locker 221/222 did not alter the accumulation of lipid droplets demonstrated by oil red O staining measured by absorbance at 490 nm (**Figure 5A**). Similarly, pLV 221, pLV 222, and pLV221/222 only show slight increase in the accumulation of lipid droplets in 3T3-L1 cells without statistical significance (**Figure 5B**). Western blot analysis demonstrated that the expression of C/EBPα and PPARγ were not changed by pLV-locker 221 and pLV-locker 222 (**Figure 5C**), while they were significantly up-regulated by treatment with pLV 222 (**Figure 5D**). To investigate lipolysis, lentiviral vectors were transduced to the fully differentiated 3T3-L1 adipocyte and we performed glycerol assay 7 days after lentiviral transduction. pLV-locker 221/222 and pLV 221/222 did not alter the glycerol release (**Figure 5E**).

## Discussion

The adipocyte-specific inhibition of *Mir221/Mir222* expression protected the mice fed with HFHS chow from the obesity. We demonstrated that the direct target of miR-221-3p and miR-222-3p was *Ddit4* and the inhibition of *Mir221/Mir222* expression resulted in upregulation of DDIT4, inactivation of AKT, activation of TSC2 and subsequent inactivation of S6K, the latter is a major target of mTORC1. The lentivirus-mediated transfer of *Mir222* gene promoted the adipogenesis demonstrated by up-regulation of C/EBPα and PPARγ in differentiated 3T3-L1 cells. We concluded that the inhibition of miR-221-3p and miR-222-3p and DDIT4-mediated inhibition of mTORC1 signaling is one of the major mechanisms for the protection from diet-induced obesity. The role of *Mir221* in adipogenesis has been focused by series of investigation and Ahonen et al. reported miR-221-3p overexpression in Simpson-Golabi-Behmel syndrome (SGBS) preadipocytes inhibited *de novo* lipogenesis and adipogenesis (18). In our investigation, overexpression by pLV 221 did not alter the lipogenesis and adipogenesis in 3T3-L1 cells; however, pLV 222 enhanced the adipogenesis. In bovine adipocyte differentiation, long non-coding RNA (lncRNA), *MIR221HG*, significantly increased adipocyte differentiation associated with dramatic increment of PPARγ (11). We also cloned mouse *Mir221hg* with 1,537 bp and identified that 1,054 bp of 5’-flanking region was deleted in *Mir221/222AdipoKO* mice. The expression of *Mir221hg* is barely detected in brown and WATs in wild type mice and we speculated that the influence of deletion of *Mir221hg* is minimal in *Mir221/222AdipoKO* mice. Although the functional studies to elucidate the role of *Mir222* in adipogenesis and lipogenesis were not reported in cultured adipocytes in previously published studies, we demonstrated that *Mir222* expression was upregulated in WATs of obese mice and in serum of obese patients. The importance of *Mir222* in the pathogenesis of obesity is supported by the analysis of adipose tissue-specific Dicer knockout mice, in which gonadal adipose tissue was the main source of serum exosomal miR-222 (19). The exosome-packed *Mir222* influenced remote organs, and overexpression of *Mir222* inhibits the expression of IRS-1 by directly binding to untranslated regions in the muscle (20) and liver (21). In current investigation, we firstly demonstrated that overexpression of *Mir222* promoted the adipogenesis in 3T3-L1 cells.

Under the presence of amino acids, mTORC1 is activated by GTP-bound Rheb. In the upstream of the Rheb, AKT inhibits TSC2 and TBC1D7 (TBC1 Domain Family Member 7) complex, which further inhibits the activation of mTORC1 by acting as a GTPase-activating protein. mTORC1 promotes the lipid synthesis and storage and adipogenesis, while it inhibits the lipolysis, β-oxidation and ketogenesis. SREBP (sterol regulatory element-binding protein) processing and activation is promoted by mTORC1 by the activation of S6K and lipin 1 leading to transcriptional activation of SREPB1, SREBP2, and many other lipogenic genes. mTORC1 also committed mesenchymal stem cells to adipocyte lineage by the activation of S6K, promotes the initial step of adipocyte differentiation by inhibiting 4E-BP1/2 (eIF4E-binding protein 1/2), and completes the final differentiation by the activation of PPARγ (22). Many researchers screened the agents or intrinsic factors to inhibit mTORC1 signaling and lipogenesis for the treatment of obesity. Lee et al. reported that ezetimibe reduced lipid accumulation by inhibiting mTORC1 signaling, leading to the downregulation of lipogenesis-related genes (23). Shi et al. reported that the inhibition of miR-196b-5p blocked adipogenesis and lipogenesis by directly targeting TSC1 and TGFBR1 (transforming growth factor-β receptor 1) (24). We demonstrated that the inhibition of miR-221-3p and miR-222-3p and subsequent DDIT4-mediated inhibition of mTORC1 signaling is a therapeutic target for the treatment of obesity.

The activation of mTORC1 links to various pathological processes in adipose tissues such as inflammation, beige adipogenesis and angiogenesis. mTORC1 loss and gain of function studies in macrophages resulted in amelioration and exacerbation of inflammatory response, as well as macrophage polarization to both M1 and M2 profiles (25). miR-221 mediates M1 macrophage polarization (26, 27), while suppression of miR-222 alleviate the inflammatory response (28, 29). However, we did not observe the apparent changes in gene expression of cytokines such as *Il1b*, *Il6*, *Ifng*, and *Tnf* in adipose tissues in *Mir221/222AdipoKO* mice (**Supplementary Figure 2**). The phenotyping of T cells and macrophages in WATs should be performed in the future investigation. In beige adipogenesis, the adipose-specific deletion of Raptor, a key component of mTORC1, promoted beige adipogenesis by inhibiting prostaglandins (PGs) synthesized by cyclooxygenase-2 (COX-2) (30). Supporting this notion, ketoprofen alleviated diet-induced obesity and promotes the fat browning by the COX-2 and mTORC1-p38 signaling pathway (31). In our investigation, the genes related to beige adipogenesis such as *Ppargc1* and *Prdm16* tended to be upregulated in WATs without statistical differences. However, in WATs in *Mir221/222AdipoKO* mice, apparent beige adipogenesis was not observed by histological examinations. In tumor microenvironment and angiogenesis, mTORC1 under a hypoxic condition promotes the translation of hypoxia-inducible factor (HIF) 1-2, which lead to the expression of angiogenic growth factors such as vascular endothelial growth factor (VEGF), TGF-α, and platelet-derived growth factor β (PDGF-β) (32). *Mir221* and *Mir222* demonstrated the angiogenic activities in vascular cells (33) and cancers (34), however, we did not observe the angiogenesis activities in WATs in *Mir221/222AdipoKO* mice.

## Limitation of Study

We hypothesized that circulating miR-221-3p and miR-222-3p play important roles in both mouse obese models and the patients with obesity and T2D. However, the circulating levels of miR-221-3p and miR-222-3p were not altered in *Mir221/222^flox/y^* and *Mir221/222AdipoKO* mice fed with STD and HFHS. In addition, the human serum levels of miR-221-3p and miR-222-3p showed correlations with HbA1c, however, they did not show significant correlations with body weight and BMI. Although we show the roles of miR-221-3p and miR-222-3p in the adipocytes, we did not clearly demonstrate the role of circulating miR-221-3p and miR-222-3p in the pathogenesis of obesity. Another limitation is that it is still unknown why *Mir221/222AdipoKO* mice did not show significant improvement of insulin sensitivity although the body weight was significantly reduced compared with *Mir221/222^flox/y^* fed with HFHS. Finally, the micronutrient composition is not matched between the STD and HFHS diets and it may possibly impact the observed outcomes. Ideally, a matched diet should be used for mineral and vitamin mix.

## Data Availability Statement

The datasets presented in this study can be found in online repositories. The names of the repository/repositories and accession number(s) can be found in the article/**Supplementary Material**.

## Ethics Statement

The studies involving human participants were reviewed and approved by Okayama University Graduate School of Medicine, Dentistry and Pharmaceutical Sciences and Okayama University Hospital, Ethics Committee. The patients/participants provided their written informed consent to participate in this study. The animal study was reviewed and approved by the Animal Care and Use Committee of the Department of Animal Resources, Advanced Science Research Center, Okayama University.

## Author Contributions

SY, DZ, AK, and JW designed the project and experiments and wrote the manuscript. SY, DZ, AK, NK, RS, and HA performed animal experiments and analyzed and interpreted data. AN and JE performed culture and molecular biology experiments. SY, AK, NK, RS, AN, JE, and JW designed and performed clinical study. All authors contributed to the article and approved the submitted version.

## Funding

This work was supported by Grant-in-Aid for Young Scientists (19K17984), Grant-in-Aid for Scientific Research (B) (19H03675), Japan Agency for Medical Research and development (AMED, grant no: 17ek0210095h0001, 20ek0109445h0001).

## Conflict of Interest

JW receives speaker honoraria from Astra Zeneca, Daiichi Sankyo, Novartis, Novo Nordisk Pharma, Tanabe Mitsubishi and receives grant support from Astellas, Baxter, Bayer, Chugai, Dainippon Sumitomo, Kyowa Kirin, Novo Nordisk Pharma, Ono, Otsuka, Tanabe Mitsubishi, and Teijin.

The remaining authors declare that the research was conducted in the absence of any commercial or financial relationships that could be construed as a potential conflict of interest.

## Publisher’s Note

All claims expressed in this article are solely those of the authors and do not necessarily represent those of their affiliated organizations, or those of the publisher, the editors and the reviewers. Any product that may be evaluated in this article, or claim that may be made by its manufacturer, is not guaranteed or endorsed by the publisher.
