# Supplementary material for: Adipocyte-Specific Inhibition of Mir221/222 Ameliorates Diet-Induced Obesity Through Targeting Ddit4
Source: Front Endocrinol (Lausanne). 2022 Jan 3;12:750261. doi: 10.3389/fendo.2021.750261 (PMC8762293; doi:10.3389/fendo.2021.750261)
Supplement: Supplementary file 1 [file DataSheet_1.pdf]

Supplementary Figure 1

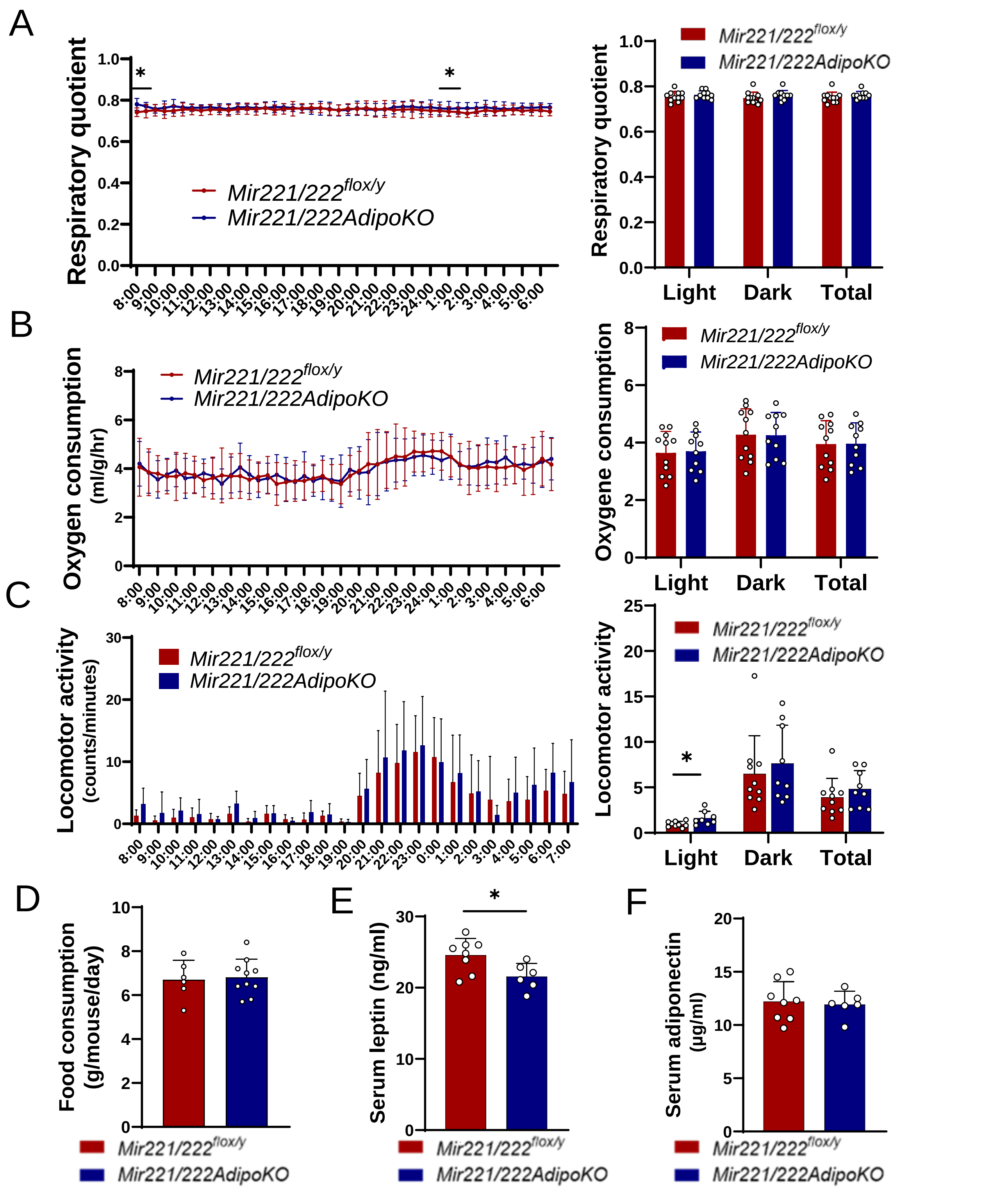

Supplementary Figure 1. Basal metabolic rate, locomotor activity and food intake. (**A and B**) Respiratory quotient (RQ) and Oxygen consumption rate in *Mir221/222$^{flox/y}$* (n=11) and *Mir221/222AdipoKO* (n=10) mice fed with HFHS (high fat high sucrose) chow. (**C**) Locomotor activity in *Mir221/222$^{flox/y}$* (n=10) and *Mir221/222AdipoKO* (n=9) mice fed with HFHS chow. (**D**) Food consumption in *Mir221/222$^{flox/y}$* (n=5) and *Mir221/222AdipoKO* (n=10) mice fed with HFHS chow. (**E**) Serum leptin levels in *Mir221/222$^{flox/y}$* (n=8) and *Mir221/222AdipoKO* (n=6) mice fed with HFHS chow. (**F**) Serum adiponectin levels in *Mir221/222$^{flox/y}$* (n=8) and *Mir221/222AdipoKO* (n=6) mice fed with HFHS chow. Data shown as mean ± SD and analyzed by Mann-Whitney's U test (*$p<0.05$).

Supplementary Figure 2

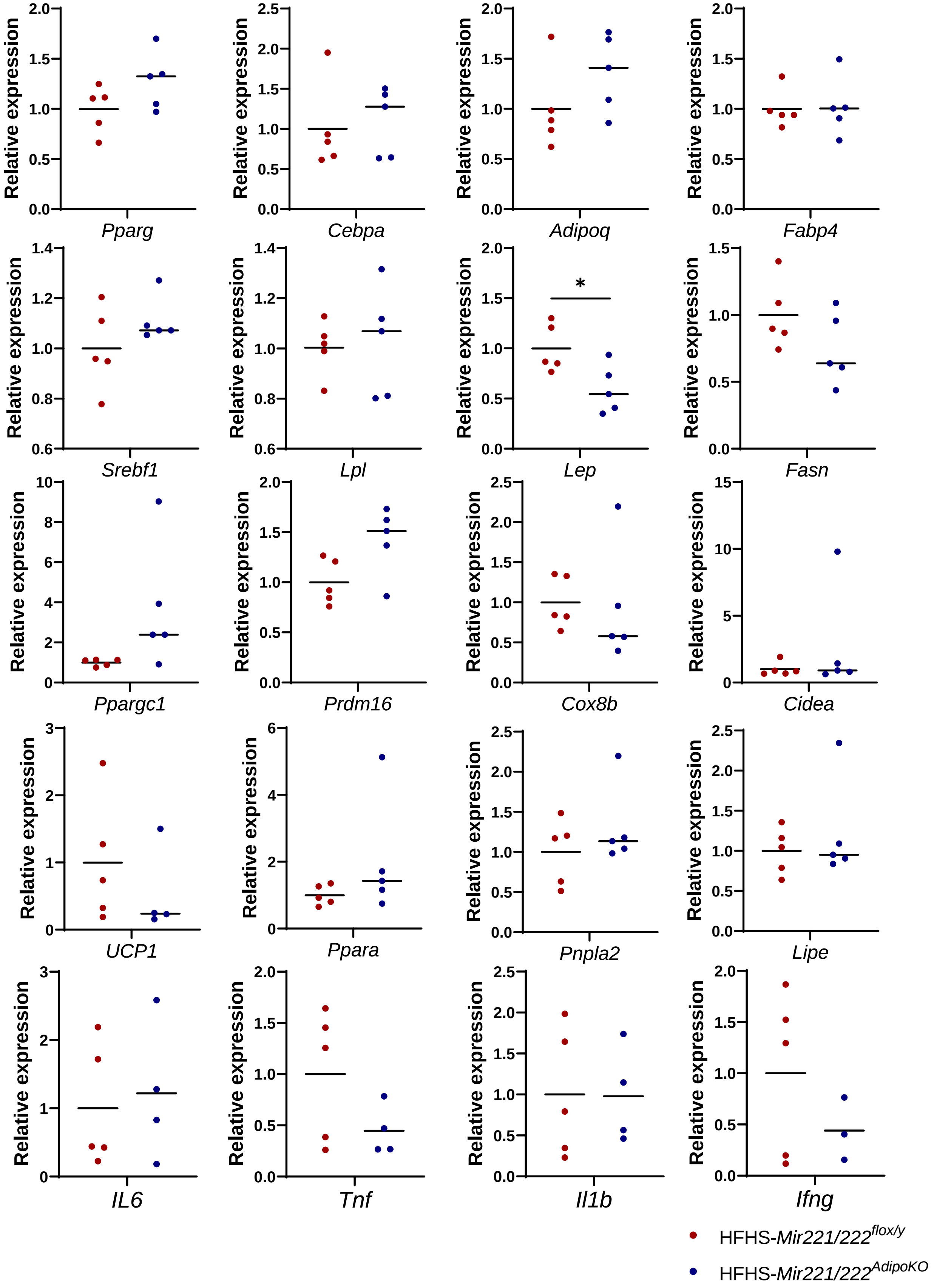

Supplementary Figure 2. mRNA expression of various genes. *Mir221/222*$^{flox/y}$ (n=5) and *Mir221/222AdipoKO* (n=5) mice fed with HFHS (high fat high sucrose) chow. Data shown as mean ± SD and analyzed by Mann-Whitney's U test (*p<0.05).

Supplementary Figure 3

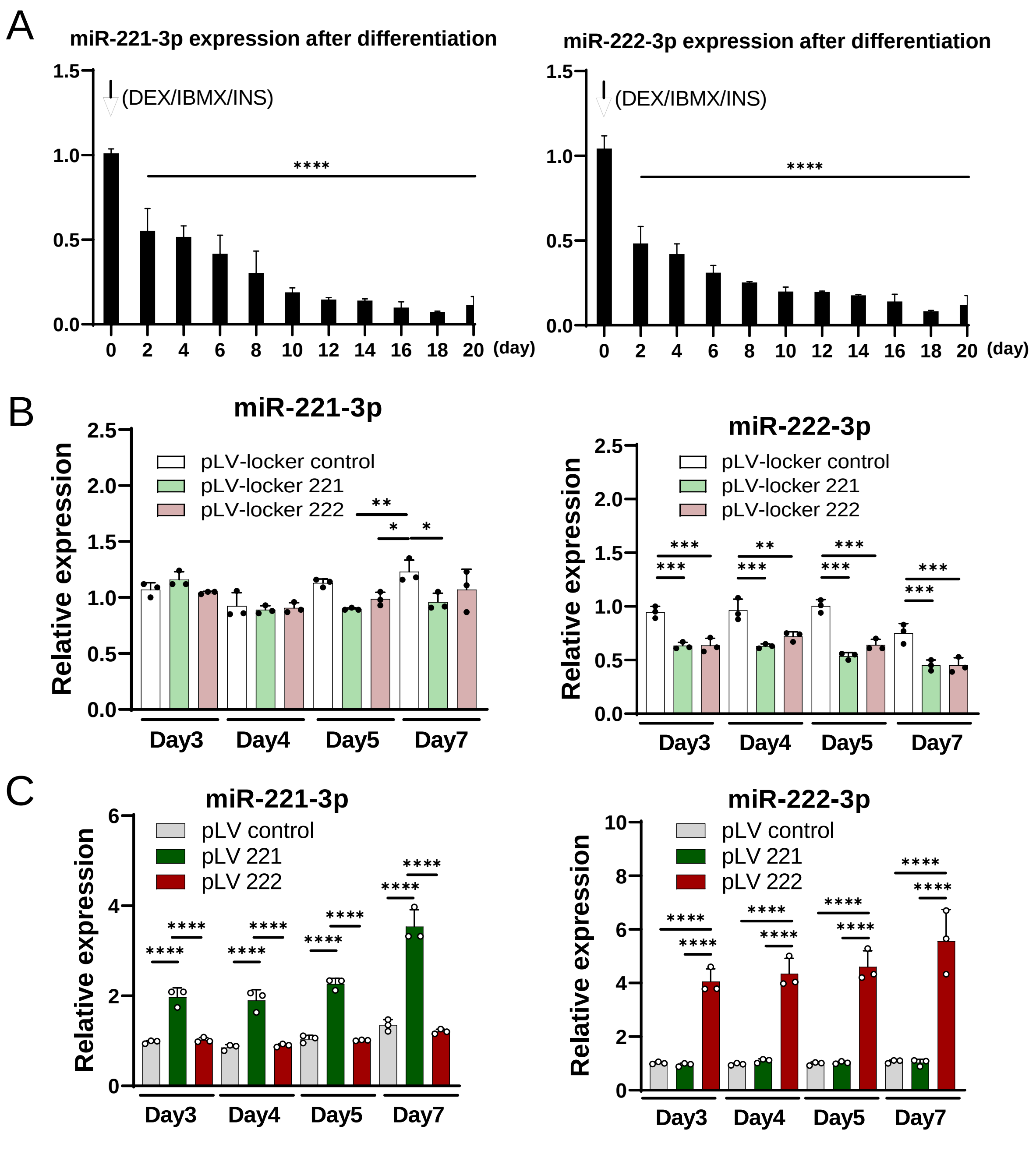

Supplementary Figure 3. Expression of miR-221-3p and miR-222-3p in 3T3-L1 cells. (**A**) Expression of miR-221-3p and miR-222-3p after the induction of adipocyte differentiation by dexamethasone, isobutylmethylxanthine, 1-methyl-3-isobutylxanthine and insulin (DEX/IBMX/INS). (n=3) (**B**) Expression of miR-221-3p and miR-222-3p in 3T3L1 cells treated with pLV-locker control, pLV-locker 221, and pLV-locker 222. (**C**) Expression of miR-221-3p and miR-222-3p in 3T3-L1 cells treated with pVL control, pLV 221, and pLV 222. Data shown as mean ± SD and analyzed one-way ANOVA with Tukey test (*p<0.05; **p<0.01; ***p<0.001; ****p<0.0001).

Supplementary Figure 4

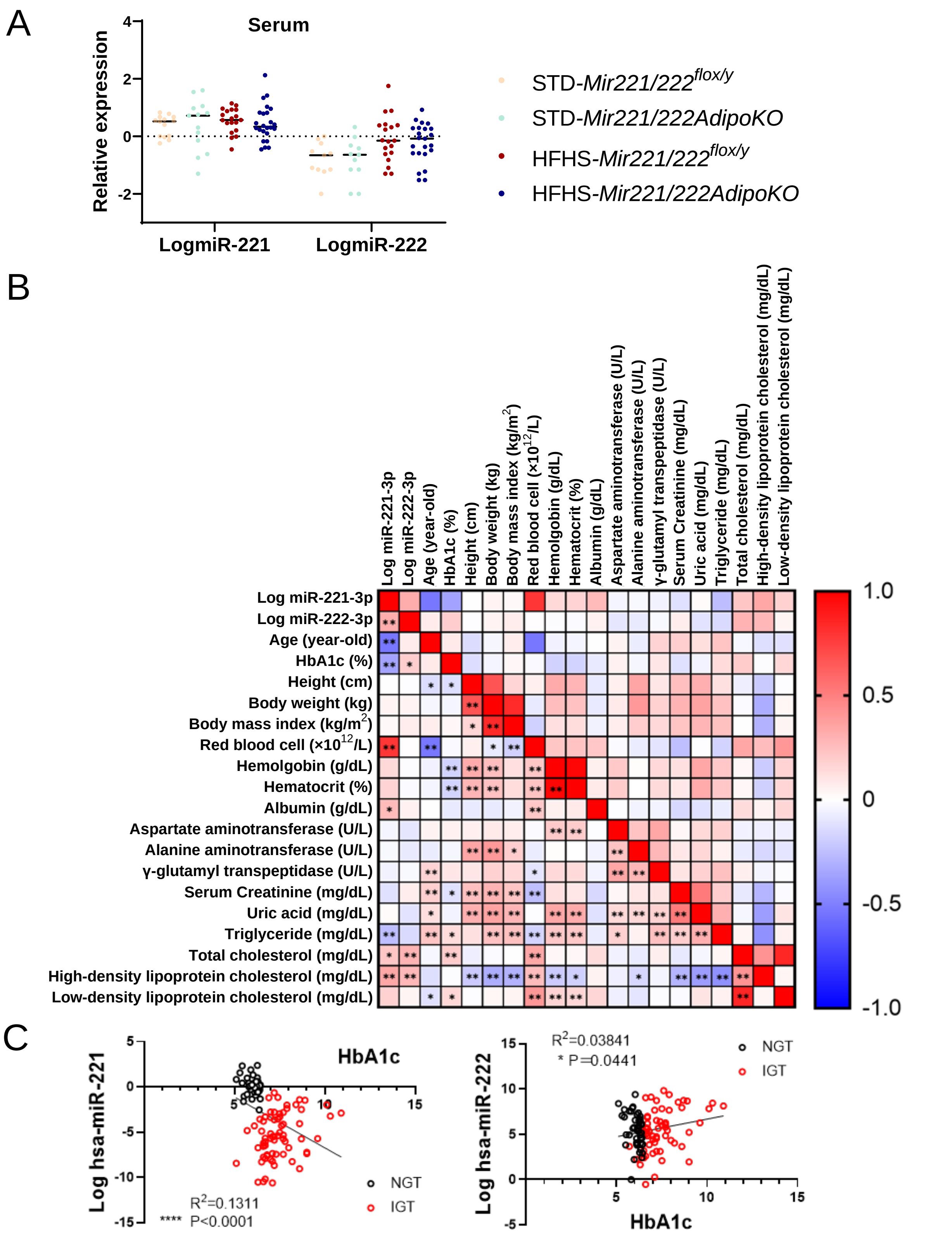

Supplementary Figure 4. Serum concentration of mir-221-3p and mir-222-3p levels in mice and human. (**A**) Serum concentrations of mir-221-3p and mir-222-3p levels in *Mir221/222*$^{flox/y}$ (n=14) and *Mir221/222AdipoKO* (n=13) mice fed with STD (standard) chow, *Mir221/222*$^{flox/y}$ (n=19) and *Mir221/222AdipoKO* (n=24) mice fed with HFHS (high fat high sucrose) chow . (**B**) Simple correlations of serum miR-221-3p and miR-222-3p levels with various clinical parameters in the subjects with normal glucose tolerance (NGT, n=45) and impaired glucose tolerance (IGT, n=69) (n=114). In correlation matrix, Spearman's rank correlation coefficient is shown. *p<0.05, **p<0.01. (**C**) Log hsa-miR-221-3p negatively correlates with HbA1c, while Log hsa-miR-222-3p positively correlates with HbA1c.

Supplementary Figure 5

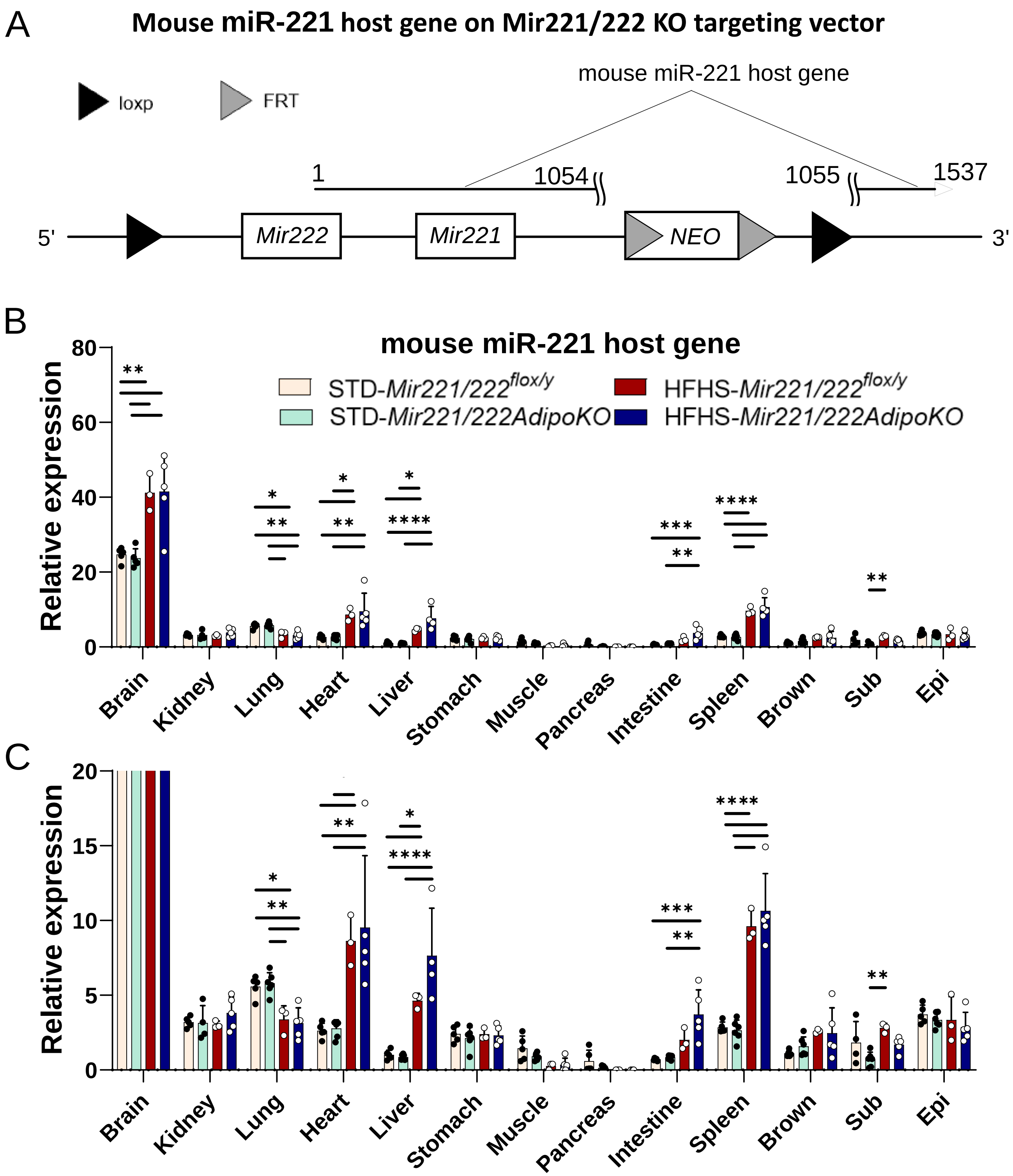

Supplementary Figure 5. Expression of miR-221 host gene (*Mir221hg*) in various tissues. (**A**) Mir221/222 KO targeting vector map and location of miR-221 host gene (*Mir221hg*). The single exon of *Mir221hg* is disrupted by NEO. (**B and C**) Expression of *Mir221hg* in various tissues in *Mir221/222*$^{flox/y}$ (n=5) and *Mir221/222AdipoKO* (n=6) mice fed with STD (standard) chow, *Mir221/222*$^{flox/y}$ (n=3) and *Mir221/222AdipoKO* (n=5) mice fed with HFHS (high fat high sucrose) chow. Whole range of relative expression (0 to 80) is shown in panel **B** and narrow range (0 to 20) in panel **C**. Data shown as mean ± SD and analyzed by one-way ANOVA with Tukey test. (*p<0.05; **p<0.01; ***p<0.001; ****p<0.0001).

Supplementary Figure 6

Uncropped images in Figure 3B

DDIT4

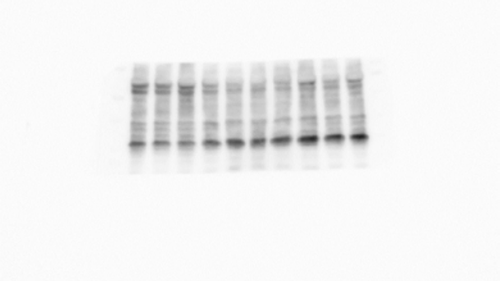

GAPDH

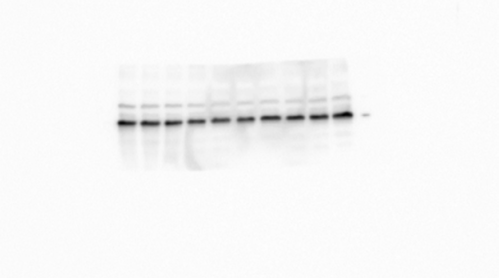

Uncropped images in Figure 4A

p-AKT

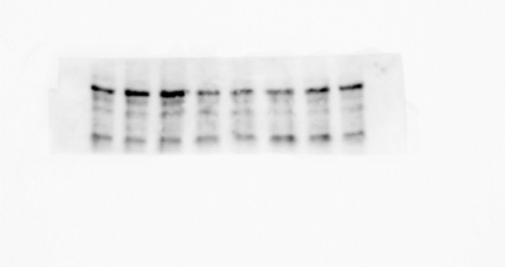

total-AKT

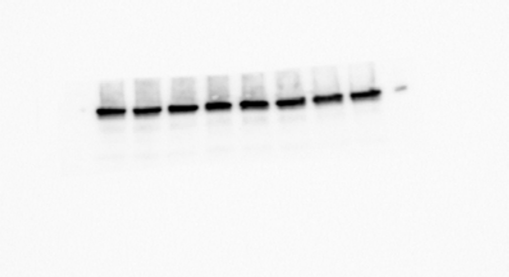

p-TSC2

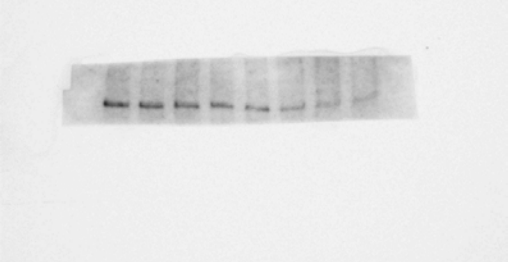

total-TSC2

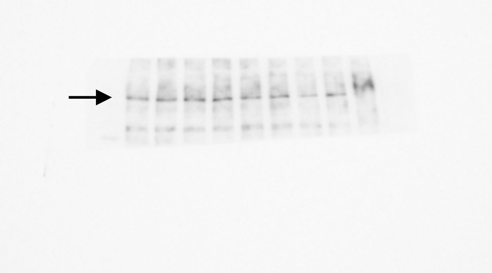

p-mTOR

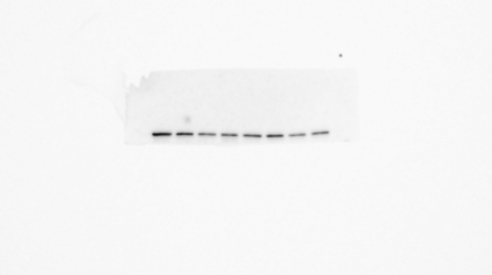

total-mTOR

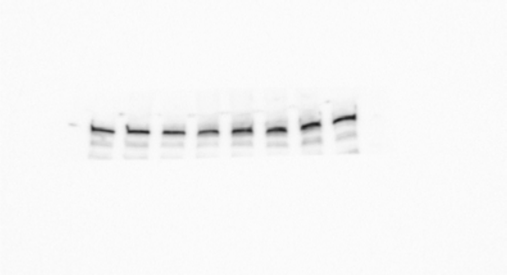

Supplementary Figure 7

Uncropped images in Figure 4A

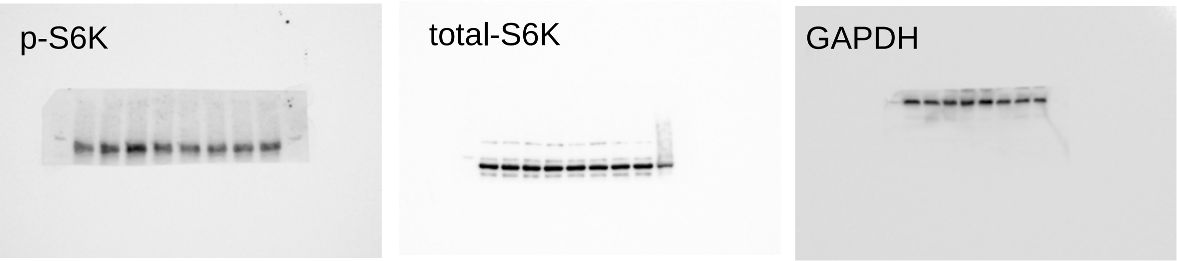

Uncropped images in Figure 5C,D

C

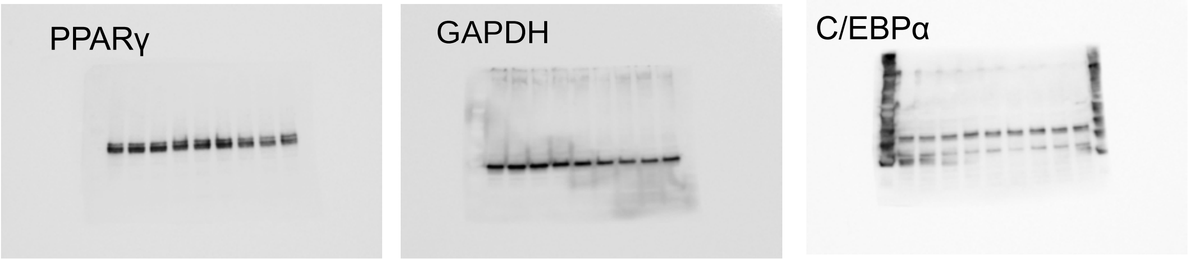

D

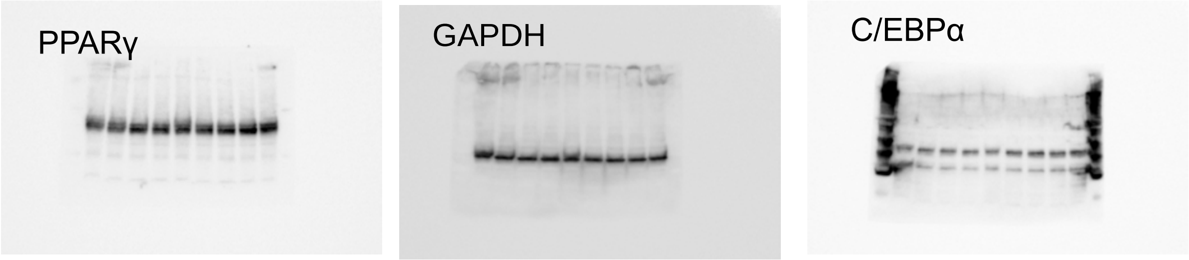

Supplementary Figure 8

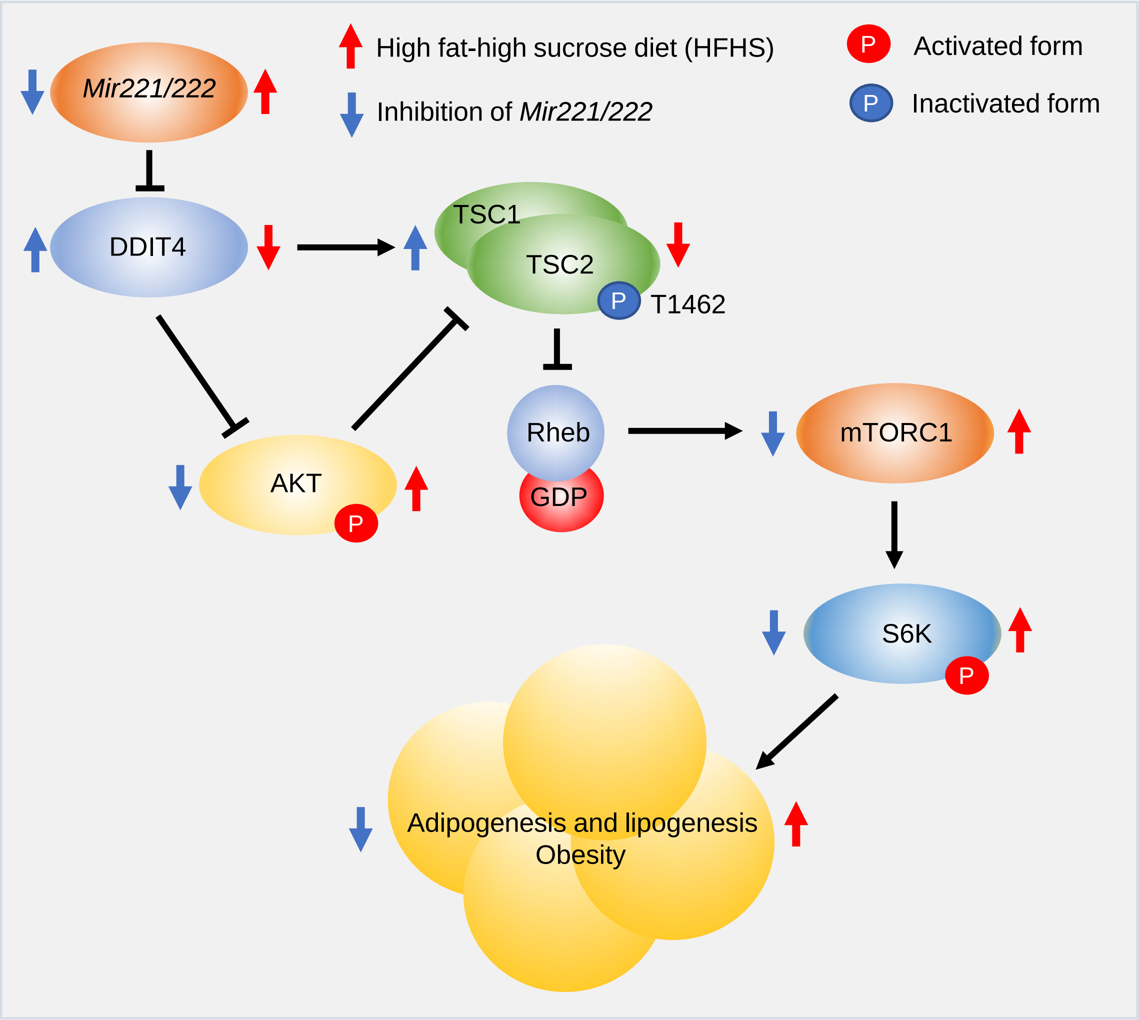

Supplementary Figure 8. Ddit4 is a direct target of miR-221-3p and miR-222-3p and it inhibits mTORC1 pathway.
